# Angioedema quality of life questionnaire (AE-QoL) - interpretability and sensitivity to change

**DOI:** 10.1186/s12955-019-1229-3

**Published:** 2019-10-26

**Authors:** Kanokvalai Kulthanan, Leena Chularojanamontri, Chuda Rujitharanawong, Puncharas Weerasubpong, Marcus Maurer, Karsten Weller

**Affiliations:** 1 Department of Dermatology, Faculty of Medicine Siriraj Hospital, Mahidol University, Bangkok, Thailand; 2 Dermatological Allergology, Allergie-Centrum-Charité, Department of Dermatology and Allergy, Charité - Universitätsmedizin Berlin, Berlin, Germany

**Keywords:** Angioedema, Quality of life, Questionnaire, Clinimetric properties, Validity, Reliability, Interpretability, Patient reported outcome

## Abstract

**Background:**

The Angioedema Quality of Life (AE-QoL) is the first patient reported outcome measure developed for the assessment of quality of life (QoL) impairment in patients with recurrent angioedema (RAE). This study aimed to evaluate the clinimetric properties of the AE-QoL in Thai patients and to establish categories of QoL impairment assessed by the AE-QoL.

**Methods:**

The validated Thai version of the Dermatology Life Quality Index (DLQI) and Patient Global Assessment of Quality of Life (PGA-QoL) were used to comparatively evaluate the Thai version of AE-QoL. Spearman correlations between the Thai AE-QoL and two other standard measurements (DLQI and PGA-QoL) were investigated to determine convergent validity. The Thai DLQI and PGA-QoL were used to categorize patients according to their QoL. Known-group validity of the Thai AE-QoL was later analyzed. The reliability of the Thai AE-QoL was investigated using Cronbach’s alpha and intraclass correlation. Three different approaches including the distribution method, receiver operating characteristic curve analysis, and the anchor based-method were used for the interpretability.

**Results:**

A total of 86 patients with RAE with a median age of 38.0 ± 15.1 years (range 18–76) were enrolled. Of those, 76 patients (88%) had RAE with concomitant wheals, and 10 patients (11.6%) had RAE only. The AE-QoL assessed RAE-mediated QoL impairment with high convergent validity and known-groups validity, high internal consistency and test-retest reliability, and good sensitivity to change. Although the AE-QoL did not differentiate between patients with moderate and large effect as measured by PGA-QoL or DLQI in this study, AE-QoL total values of 0–23, 24 to 38, and ≥ 39 could define patients with “no effect”, “small effect”, and “moderate to large effect” of RAE on their QoL, respectively.

**Conclusions:**

This study supports the validity and reliability of the Thai version of the AE-QoL, which is a very different language from the original version. Categories allow to classify the effect of RAE on patients’ QoL as “none”, “small”, and “moderate to large”. Further studies are needed to confirm the applicability of AE-QoL in other Asian populations”.

## Background

Recurrent angioedema (RAE) is characterized by the unpredictable, sudden and recurrent onset of nonpitting swelling of the skin, subcutaneous tissues, and/or mucous membranes [1]. Angioedema attacks may be painful, disabling, and disfiguring depending on their duration, location and the extent of swelling. Swellings in RAE patients can cause itch or a burning sensation, often they do not. Cases of RAE with upper airway involvement can be an emergency and life-threatening condition.

RAE is classified into mast cell mediator-mediated and bradykinin-mediated, based on the underlying pathogenic mechanisms [2]. In clinical practice, RAE most commonly occurs in chronic spontaneous urticaria (CSU) patients, with or without wheals [3]. RAE is associated with markedly impaired health-related quality of life (HRQoL) [4, 5]. Effective management of RAE requires the use of angioedema specific instruments designed to evaluate patients’ impairment in HRQoL.

For assessing HRQoL of angioedema patients, the Angioedema Quality of Life Questionnaire (AE-QoL) [6] and the Hereditary Angioedema Quality of Life Questionnaire (HAE- QoL) have been developed [7–9]. The HAE-QoL assesses HAE-specific effects on QoL, whereas the AE-QoL was developed for the use in patients with all forms of RAE including HAE. The original German version of the AE-QoL consists of 17 questions in 4 domains (functioning, fatigue/mood, fear/shame, and food) that collectively evaluate the extent of RAE-dependent QoL impairment during the previous 4 weeks. Each AE-QoL question has 5 answer options (scored 1–5), with lower and higher scores indicting less and more adverse impact, respectively. The total score is calculated, which is then transformed into a linear scale that ranges from 0 to 100, with a score of 100 indicating the worst possible impairment of HRQoL [6]. The AE-QoL was shown to be valid and reliable for use in clinical practice and has been used in multiple randomized controlled trials [10–15]. The minimal clinically important difference (MCID), which is defined as the least difference in a score that patients can notice the improvement in their HRQoL, was proposed to be > 6 points [6]. The AE-QoL has been translated and culturally adapted for the use in 25 countries in 29 languages [16].

As of now, there is the lack of and need for categorization/banding of HRQoL impairment in RAE patients. Also, we are lacking validated version of patient-reported outcome (PRO) outcome measures for assessing QoL impairment in Asian patients with RAE. Accordingly, this study generated the Thai version of the AE-QoL, evaluated its clinimetric properties in an Asian population of RAE patients, specifically in Thailand, and established categories of AE-QoL-assessed QoL impairment in this RAE population.

## Methods

### Generation of the Thai AE-QoL

Formal permission was given by MOXIE GmbH (moxie-gmbh.de/, Berlin, Germany) to translate the original German version of the AE-QoL into Thai, and to validate the translated instrument [16]. The AE-QoL were independently translated into Thai by two native Thai speakers with command of the German language. Both Thai versions were then reviewed and revised by dermatologists with subspecialty expertise in allergy with the objective of achieving unanimous agreement for each item. The final Thai version of each instrument was then independently back-translated into German by two bilingual translators. The two back-translated German versions were then reviewed by the German developers of the original instrument.

Misconceptions or misinterpretations during the translation process were resolved via a cooperative review and editing process that included the Thai research team and the instrument developers. After consensus was reached among all assessors, cognitive debriefing was performed with representatives of the target population and target language group to evaluate if the responders understand the questionnaire as intended [17, 18]. The final Thai version of the AE- QoL was tested in 10 Thai RAE patients. This test revealed no points of contention or misinterpretation. The final Thai versions of the AE-QoL was then approved and used in this study.

### Patients and conduct of study

This study was approved by the Siriraj Institutional Review Board (SIRB), Faculty of Medicine Siriraj Hospital, Mahidol University, Bangkok, Thailand (COA no. Si271/2017). Adult Thai RAE patients aged 18 years or older attending the Urticaria Center of Reference and Excellence [19] of the Department of Dermatology, Siriraj Hospital during May 2017 to May 2018 were invited to voluntarily participate. Siriraj Hospital is Thailand’s largest national tertiary referral center. Patients with RAE were defined as patients with the unpredictable, sudden and recurrent onset of nonpitting swellings of the skin, subcutaneous tissues, and/or mucous membranes for longer than 6 weeks [1]. Patients who had literacy difficulties, dermatologic diseases other than RAE, and/or mental diseases were excluded. Standard treatment for RAE was given to all patients regardless of their participatory status in this study. This study complied with the principles set forth in the Declaration of Helsinki (1964) and all of it subsequent amendments, and written informed consent was obtained from all participating patients.

### Patient HRQoL questionnaires used to compare with the Thai version of the AE-QoL

#### The validated Thai version of the dermatology life quality index (DLQI)

The DLQI is a general dermatologic HRQoL questionnaire that was established by Finlay et al. in 1994 [20]. The DLQI consists of 10 items, with a minimum and maximum score of 0 and 30, respectively. Total DLQI scores are categorized into one of the following five groups: 0–1 = no effect, 2–5 = small effect, 6–10 = moderate effect, 11–20 = very large effect, and 21–30 = extremely large effect. The Thai version of the DLQI was used [21].

#### Patient global assessment of quality of life (PGA-QoL)

The PGA-QoL is a patient evaluation instrument for assessing HRQoL during the preceding four-week period. A 5-point Likert-scale is used to rate level of effect, as follows: 0 = no effect, 1 = mild effect, 2 = moderate effect, 3 = severe effect, and 4 = very severe effect [22, 23].

At baseline, patients were informed how to complete the following questionnaires: Thai AE-QoL, Thai DLQI, and PGA-QoL. After patients acknowledged understanding of how to complete all three questionnaires, they were asked to complete the Thai AE-QoL, Thai DLQI, and PGA-QoL by themselves at the clinic. After 4 weeks of treatment (2nd visit), all of the questionnaires were once again completed by the patients in the clinic.

### Statistical analysis

Consensus-based Standards for the Selection of Health Measurement Instruments (COSMIN), a standard protocol for evaluating the methodologic quality of studies in health measurement instruments, was applied in this study [24]. The Statistical Package for the Social Sciences for Windows, Version 18.0 (SPSS Inc., Chicago, IL, USA) was used to analyze data. *P*- values of less than or equal to 0.05 were considered to be statistically significant.

### Assessment of AE-QoL validity

Convergent validity indicates the correlation between the Thai AE-QoL and other standard measurements for HRQoL (DLQI and PGA-QoL). Spearman correlation coefficients of < 0.3, 0.3–0.6, and > 0.6 were considered weak, moderate, and strong correlations, respectively [25].

Known-group validity demonstrates the ability of the AE-QoL to discriminate groups that are assumed to be different. For HRQoL, patients were classified into three groups using the DLQI and PGA-QoL scores in this study, as follows: (i) ‘no effect’ (DLQI scores of 0–1, PGA- QoL score of 0); (ii) ‘small effect’ (DLQI scores of 2–5, PGA-QoL score of 1); and, (iii) ‘moderate to large effect’ (DLQI scores of 6–30, PGA-QoL scores of 2–4).

### Assessment of AE-QoL reliability

Cronbach’s α reliability coefficient was used to analyze internal consistency. Excellent, good, and acceptable reliability were defined as α values of ≥0.9, 0.7 ≤ α < 0.9, 0.6 ≤ α < 0.7, respectively [26]. Internal consistency determines the homogeneity of AE-QoL domains, which can indicate whether it is appropriate to calculate a total score. The four domain scores of the AE-QoL were investigated for internal consistency in each domain.

Test-retest reliability assesses the consistency of scores across multiple administrations. Stable patients should have comparable scores at two different administrations. Data from patients with no change in PGA-QoL score (stable in HRQoL) during 4 weeks of treatment was used to determine test-retest reliability. Intraclass correlation coefficient (ICC) values of < 0.40, 0.4–0.75, and > 0.75 indicate poor, average, and strong reliability, respectively [27].

### Assessment of AE-QoL sensitivity to change

Sensitivity to change is defined as the ability of an instrument to detect change over time, regardless of whether the change is meaningful [28]. Correlation coefficients of 0.1–0.3, 0.3–0.5, and > 0.5 were considered weak, moderate, and large correlations, respectively [29]. At least moderate correlation between an instrument and a standard comparative measure was required to further analyze AE-QoL interpretability in this study.

### Assessment of AE-QoL interpretability

Interpretability is the ability of an instrument to transform qualitative meaning to quantitative scores, i.e. the degree to which the values obtained by the use of the instrument produce information relevant to the patient and the physician in relation to the measured construct. Interpretability is composed of the minimal clinically important difference (MCID) and categorization. The MCID is defined as the smallest difference in a score that a patient can recognize as meaningful improvement [30, 31]. Different approaches were used to define the MCID are described, as follows:

i. Distribution method (MCID-1) indicates the numerical distribution of values. The standard error of measurement (SEM) is widely accepted to represent the MCID of an instrument [32–34]. The calculation of SEM was (1-reliability of the Thai AE-QoL)1/2 × the SD of the Thai AE-QoL at baseline.
ii. Receiver operating characteristic (ROC) curve analysis (MCID-2) shows the sensitivity and specificity over the range of absolute reductions in scores that can be used to identify “improvement” [34, 35]. Area under the curve (AUC) values of 1, 0.9, 0.8, 0.7, and 0.5 were interpreted as perfect, excellent, good, fair, and no better than chance, respectively [36].
iii. Anchor-based approach (MCID-3) compares changes in scores to an anchor value. MCID-3 is the difference between the mean “improvement” score and the mean “worsening/stable” score. Patients with a decrease in PGA-QoL score of ≥1 and patients with an increased or unchanged PGA-QoL score were defined as ‘improvement’ and ‘worsening or stable’ HRQoL, respectively.

### Categorization of effects of RAE on quality of life

Categorization is the process of determining cutoff scores for a measurement instrument that are then used to categorize patients into different groups. The DLQI and PGA-QoL were used to categorize patients into “no effect” (DLQI = 0–1, PGA-QoL = 0), “small effect” (DLQI = 2–5, PGA-QoL = 1), “moderate effect” (DLQI = 6–10, PGA-QoL = 2), and “large and very large effect” (DLQI = 11–30, PGA-QoL = 3–4).

## Results

A total of 86 patients with RAE with a median age of 38.0 ± 15.1 years (range 18–76) were enrolled in this study. Of those, 76 patients (88%) had RAE with concomitant wheals, and 10 patients (11.6%) had RAE only. Of 10 patients who had RAE without wheals, 9 and 1 patients had idiopathic angioedema, and angiotensin-converting enzyme inhibitor-induced angioedema, respectively. Demographic data, scores at baseline, and treatments are shown in Table 1. AE- QoL scores were observed to be normally distributed. The mean AE-QoL scores at baseline was 26.6 ± 20.7.

**Table 1:** Demographic and clinical characteristics of patients with recurrent angioedema (RAE) (*n* = 86) as well as their response to treatment and quality of life impairment as assessed by use of the Thai Angioedema Quality of Life questionnaire (AE-QoL), the Thai Dermatology Life Quality Index (DLQI), the Patient Global Assessment of Quality of Life (PGA-QoL)

				Values
Gender, n (%)				
Female				69 (80.2%)
Male				17 (19.8%)
Diagnosis, n (%)				
RAE without wheals				10 (11.6%)
RAE with wheals				76 (88.0%)
Range of scores (total score)				
Thai AE-QoL				0–79.4 (100)
Thai DLQI				0–23 (30)
PGA-QoL				0–3 (4)
Disease severity of urticaria of 76 RAE patients with wheals				
Urticaria activity score over 7 days (UAS7), n (%)				
Urticaria-free (UAS7 = 0)				18 (23.7%)
Well-controlled CSU (UAS7 = 1–6)				13 (17.1%)
Mild activity CSU (UAS7 = 7–15)				26 (34.2%)
Moderate activity CSU (UAS7 16–27)				14 (18.4%)
Severe activity CSU (UAS7 = 28–42)				5 (6.6%)
Treatment, n (%)				
H1-antihistamines				74 (86.0%)
H1-antihistamines + prednisolone				8 (9.3%)
H1-antihistamines + omalizumab				4 (4.7%)
Impairment of health-related quality of life using:				
- PGA-QoL questionnaire, n (%)	AE-QoL total value mean ± SD (median)	1st quartile	3rd quartile	
None	11.2 ± 14.2 (5.9)	0	19.1	35 (40.7%)
Mild	29.9 ± 13.2 (32.4)	20.6	38.2	31 (36.0%)
Moderate	49.3 ± 17.6 (52.2)	34.9	67.3	12 (14.0%)
Severe	46.9 ± 20.4 (42.6)	34.6	61.8	8 (9.3%)
Very severe	0	0	0	0 (0.0%)
- DLQI questionnaire, n (%)				
No effect	11.6 ± 13.6 (5.9)	0	19.9	33 (38.4%)
Small effect	26.0 ± 15.3 (24.3)	16.9	34.6	22 (25.6%)
Moderate effect	39.7 ± 17.3 (38.2)	33.1	49.3	13 (15.1%)
Very large effect	42.2 ± 19 (42.6)	35.7	61.8	18 (20.9%)
Extremely large effect	0	0	0	0 (0.0%)

### The AE-QoL is a valid tool for assessing RAE-mediated QoL impairment

The AE-QoL showed high convergent validity, with strong positive correlations between AE-QoL and DLQI total score values (r = 0.72, *p* < 0.0001) and between AE-QoL total score values and PGA-QoL values (r = 0.70, *p* < 0.0001).

**Fig. 1:**
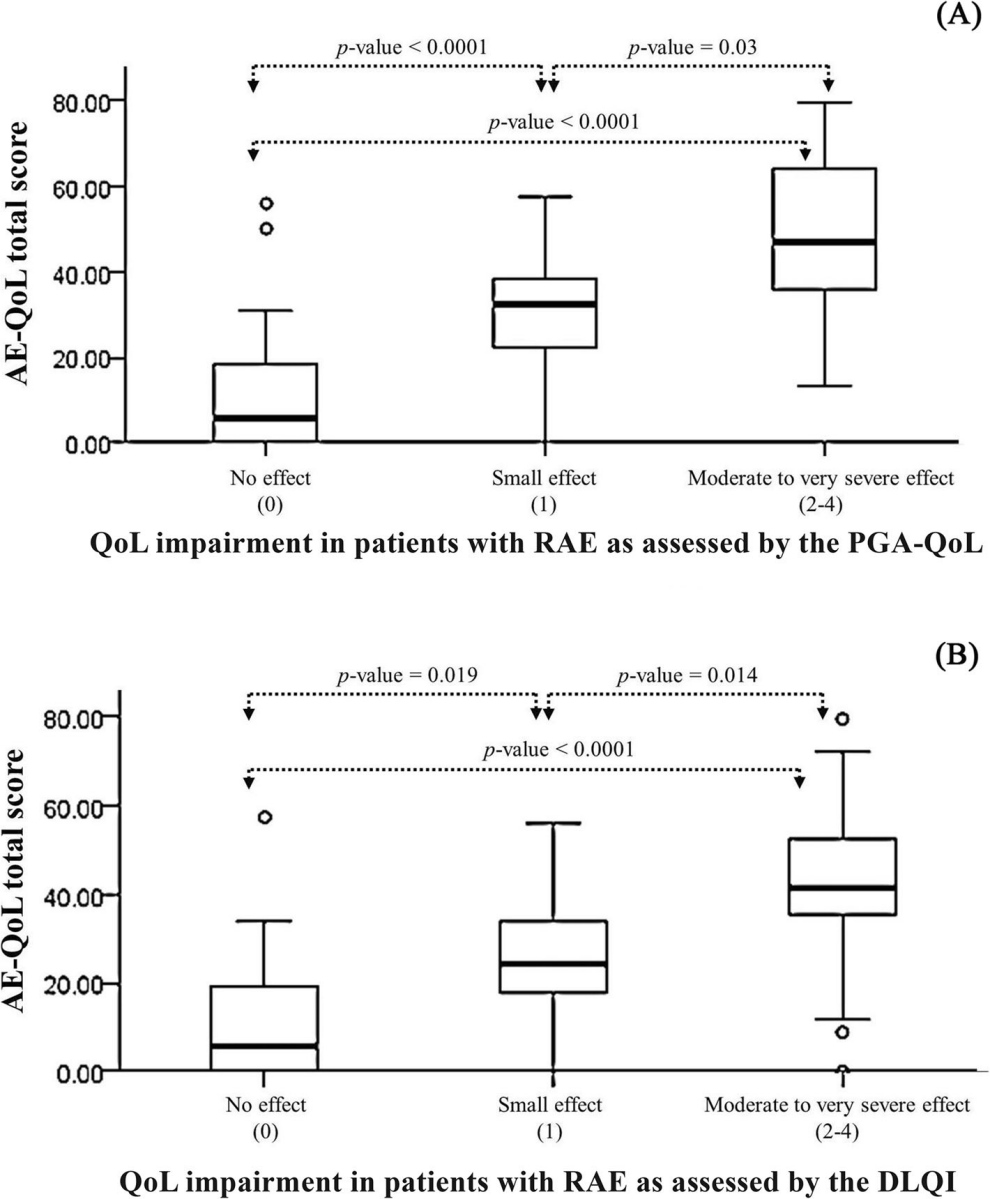
The Thai version of the Angioedema Quality of Life (AE-QoL) questionnaire can differentiate patients with recurrent angioedema (RAE) who differ in their quality of life (QoL) impairment as assessed by the use of the Patient Global Assessment of Quality of Life, PGA- QoL, (**a**) and the Dermatology Life Quality Index, DLQI (**b**)

The AE-QoL also showed high known-groups validity as it was able to discriminate among patients who showed differences in HRQoL impairment as assessed by the use of the PGA-QoL and the DLQI. Figure 1 demonstrates that the differences between the mean AE-QoL scores of patients with no effect and a small effect (*p* < 0.0001, *p* = 0.019) and with a small effect and a moderate to large effect (*p* = 0.03, *p* = 0.014) were significant. Overall, there were also significant differences in the mean AE-QoL score among patients with no effect, small effect, and moderate to large effect on HRQoL (*p* < 0.0001). However, the AE-QoL did not differentiate between patients with moderate and large effect as measured by PGA-QoL or DLQI. A statistical significance was not achieved when using the DLQI or the PGA-QoL to classify patients as those with “no effect”, a “small effect”, a “moderate effect”, and a “large effect”.

### The AE-QoL shows high internal consistency and test-retest reliability

All Cronbach’s α values were higher than 0.72, which indicates good to excellent internal consistency of the Thai AE-QoL. Forty-four patients who had no change in HRQoL (no change in PGA-QoL score) during 4 weeks were analyzed for intraclass correlation coefficients (ICCs). The ICC was 0.76 for the AE-QoL total score, which indicates strong intra-rater reliability. The ICCs (range: 0.64–0.75) for the AE-QoL domains were average (Table 2).

**Table 2:** Internal consistency of the Thai version of the Angioedema Quality of Life (AE-QoL) questionnaire

Domain	Items	Mean score (0–100)	SD	Cronbach’s α* (*n* = 86)	ICC** (*n* = 44)
Total score		26.55	20.70	0.94	0.76
Domain 1 (functioning)	1,2,3,4	20.49	23.83	0.94	0.75
Domain 2 (fatigue/mood)	6,7,8,9,10	23.66	22.33	0.86	0.64
Domain 3 (fear/shame)	12,13,14,15,16,17	35.17	28.34	0.92	0.64
Domain 4 (nutrition/food)	5,11	25.87	27.77	0.72	0.66

**Abbreviation:** SD, standard deviation; ICC, intraclass correlation coefficient

*Cronbach’s alpha values reflect analysis of data from 86 patients that completed the Thai AE-QoL questionnaire for the first time

**ICC values reflect the analysis of data from 44 patients who had stable disease (no change in Patient Global Assessment of Quality of Life questionnaire) after 4 weeks of treatment

### The AE-QoL shows good sensitivity to change

The correlation between AE-QoL score changes and changes in PGA-QoL and DLQI were 0.39 and 0.42, respectively (*p* < 0.0001). This allowed us to further analyze MCID.

The MCID-1 indicates the numerical distribution of values, The MCID-1 values using Cronbach’s α value, and ICC value were 5.07, and 10.14, respectively. For MCID-2, the sensitivity and specificity over the range of absolute reductions in scores that can be used to identify “improvement”, 30 and 56 patients were defined as ‘improvement’ and ‘worsening/stable’, respectively. An AUC of 0.81 indicated that AE-QoL is a good tool for detecting clinically meaningful improvement. Changes in AE-QoL total score of 6 and 7 had good sensitivity (73.2%) and specificity (76.7%) for defining patients with meaningful improvement in HRQoL (Table 3). For MCID-3, the difference in the mean total AE-QoL score between ‘improvement’ patients and ‘worsening/stable’ patients was 11.83.

**Table 3:** Changes in AE-QoL total score to identify “improvement” (decrease in PGA-QoL score of ≥1) and “worsening/stable” (increased or unchanged PGA-QoL score)

Changes in AE-QoL total score	Sensitivity (%) (patients correctly classified as worsening/stable)	Specificity (%) (patients correctly classified as improvement)	Accuracy (%) (proportion of correctly classified patients)
5	64.3%	76.7%	68.6%
6	73.2%	76.7%	74.4%
7	73.2%	76.7%	74.4%
8	75.0%	70.0%	73.3%
9	78.6%	70.0%	75.6%
10	78.6%	70.0%	75.6%
**AUC**	0.81 (0.72–0.91)		

Abbreviations: *AE-QoL*, Angioedema quality of life; *PGA-QoL*, Patient global assessment quality of life; *AUC*, Area under the curve

### Categorization of AE-QoL-assessed effects of RAE on QoL

The AUCs for classifying patients into the “no effect” group using PGA-QoL and DLQI were 0.88 and 0.85, respectively (Table 4). AE-QoL total scores of 23 and 25 should be used to differentiate patients between “no effect” and “small to large effect” using PGA-QoL and DLQI, respectively. To classify patients into the ‘moderate to large effect’ group, the AUC values using PGA-QoL and DLQI were 0.87, and 0.86, respectively. Using PGA-QoL and DLQI, patients with AE-QoL total scores higher than 38 and 36 would be those with ‘moderate to large effect’ on HRQoL.

**Table 4:** Cutoff values of the Angioedema Quality of Life (AE-QoL) questionnaire that differentiate patients with “no effect” from patients with “moderate to large effect” on health-related quality of life (HRQoL) using the Patient Global Assessment Quality of Life (PGA-QoL) and the Dermatology Life Quality Index (DLQI)

Patients with no effect on HRQoL using:						
	PGA-QoL score of 0			DLQI score of 0–1		
AE-QoL score	Sensitivity (%)	Specificity (%)	Accuracy (%)	Sensitivity (%)	Specificity (%)	Accuracy (%)
22	85.7%	80.4%	82.6%	78.8%	73.6%	75.6%
**23**	**88.6%**	**80.4%**	**83.7%**	81.8%	73.6%	76.7%
24	88.6%	78.4%	82.6%	84.8%	73.6%	77.9%
25	88.6%	78.4%	82.6%	**84.8%**	**73.6%**	**77.9%**
26	88.6%	74.5%	80.2%	87.9%	71.7%	77.9%
**AUC**	0.88 (0.80–0.96)			0.85 (0.77–0.94)		
Patients with moderate to large effect on HRQoL using:						
	PGA-QoL score of 2–4			DLQI score of 6–30		
AE-QoL score	Sensitivity (%)	Specificity (%)	Accuracy (%)	Sensitivity (%)	Specificity (%)	Accuracy (%)
35	78.8	75.0%	77.9%	89.1%	74.2%	83.7%
36	78.8	75.0%	77.9%	**89.1%**	**74.2%**	**83.7%**
37	81.8	75.0%	80.2%	90.9%	71.0%	83.7%
**38**	**81.8**	**75.0%**	**80.2%**	90.9%	71.0%	83.7%
39	87.9	65.0%	82.6%	92.7%	54.8%	79.1%
**AUC**	0.87 (0.78–0.95)			0.86 (0.77–0.95)		

**Abbreviation:** *AUC*, Area under the curve Bold entries indicate the most important results as discussed in the text

## Discussion

This study is the first to validate the AE-QoL in Asia. More importantly, it provides, for the first time, AE-QoL categories that define patients with “no effect”, a “small effect”, and a “moderate to large effect” of RAE on HRQoL.

We found the AE-QoL to be a valid tool for assessing and differentiating levels of HRQoL impairment in Asian patients with angioedema, as shown by the strong correlations between AE-QoL and DLQI, and between AE-QoL and PGA-QoL. It is very important for an instrument to be able to detect meaningful improvement or patient responsiveness to change. In the original version (German version) of the AE-QoL, a change in the total score of > 6 was identified as the MCID [37]. It is generally accepted that MCID values depend on the study population and the clinical context. The determination of the MCID should be based on the anchor-based method or a ROC approach rather than the distribution method as the latter relies on statistical properties and its usefulness in clinical practice is questionable. Thus, three different methods of calculating MCID have been used in this study and yielded MCIDs that ranged from 5.07 to 11.83. However, ROC curve analysis revealed that changes in AE-QoL scores of 6 or 7 had both good sensitivity (73.2%) and good specificity (76.7%) for defining meaningful change according to the judgment of the patient. We, therefore, adopted an MCID of > 6 points, which is the same as the original version, to facilitate consistency between versions in both clinical research and routine clinical practice settings.

According to the results of previous study, the linear correlation between AE-QoL total score and self-rated HRQoL impairments was well-demonstrated [6]. However, the categorization of the AE-QoL score was not established. This study provides additional information on the interpretation of the AE-QoL with regard to categorization, which is essential for the interpretation of qualitative meaning to quantitative scores [38]. In our study, only three groups of patients can be categorized; those with no effect, a small effect, and a moderate to large effect of their RAE on their QoL as a statistical significance was not achieved when using the DLQI or the PGA-QoL to classify patients as those with “no effect”, a “small effect”, a “moderate effect”, and a “large effect”. The reason is that we had a limited number of patients with severe or large effects on QoL. The prevalence of RAE varies depending on geographic regions, the prevalence of RAE was found to be low in Thailand [1]. The previous study and this study showed that HAE is uncommon in the Thai population [1]. Generally, the disease activity of HAE is more severe than that of other forms of angioedema [39]. Accordingly, our patients, on average, had less severe RAE as compared to other populations in Western countries [1, 40–44].

The maximum values to classify patients into “no effect” group were 23 and 25 using PGA-QoL and DLQI, respectively. The maximum value of 23 should be used as the cutoff score for the “no effect” category, as it had a higher AUC value than that of 25. Similarly, the minimum values to classify patients as those with a moderate to large effect were 38 and 36 using PGA-QoL and DLQI, respectively. The value of 38 should be chosen the cutoff value, as it had a higher AUC than that of 36. Thus, we propose that AE-QoL total score values from 0 to 23, from 24 to 38, and ≥ 39 define patients with “no effect”, a “small effect”, and a “moderate to large effect” of their RAE on their HRQoL, respectively. To provide a clinical perspective on this AE-QoL categorization: In the recent X-ACT study, in CSU patients with angioedema refractory to H1-antihistamine, patients had a “very large” negative impact on their HRQoL as reflected by the mean DLQI total score of 14.6 at baseline. The mean AE-QoL total score at baseline of these patients was 56.2. Using our AE-QoL categorization, the patients in this study fall into the category “moderate to large effect” of RAE on HRQoL. After 12 weeks of omalizumab treatment in this study, the mean AE-QoL total score declined significantly from 56.2 to 27.7, which changed the AE-QoL category of patients from “moderate to large effect” to “small effect” of RAE on HRQoL [13].

The strengths of this study include the use of three different approaches to establish the MCID of the Thai AE-QoL. However, several limitations should be noted including its small study population and the very high predominance of RAE patients with CSU. Secondly, no patients with HAE were enrolled in this study. Angioedema activity in HAE is generally more severe than in other causes of angioedema. Kulthanan, et al. reported that HAE was observed in 1 out of 105. Lastly, the AE-QoL did not differentiate between patients with moderate and large effect as measured by PGA-QoL or DLQI in this study. Further studies in larger study populations that include patients with high disease activity and causes of RAE other than CSU are needed to confirm the categorization of the AE-QoL.”

## Conclusion

The Thai version of AE-QoL was found to be a valid and reliable instrument for assessing disease–specific impact on QoL in Thai RAE patients. A change in AE-QoL of > 6 points defines a meaningful change in HRQoL. RAE patients should be categorized for the impact of their disease on their QoL as those with “no effect”, “small effect”, and “moderate to large effect” based on AE-QoL total score values of up to 23, 24–38, and 39 and higher, respectively.

## Data Availability

The dataset analyzed during the study is not publicly available due to required data protection, but available upon reasonable request with approval by the corresponding author.

## Abbreviations

AE-QoL: The Angioedema Quality of Life
AUC: Area under the curve
CSU: chronic spontaneous urticaria
DLQI: Dermatology Life Quality Index
HAE- QoL: Hereditary Angioedema Quality of Life Questionnaire
HRQoL: health-related quality of life
ICC: Intraclass correlation coefficient
MCID: minimal clinically important difference
PGA-QoL: Patient Global Assessment of Quality of Life
PRO: Patient-reported outcome
QoL: quality of life
RAE: Recurrent angioedema
ROC: Receiver operating characteristic
SEM: Standard error of measurement
